# Supplementary material for: Kinetics of Cellular Cobalamin Uptake and Conversion: Comparison of Aquo/Hydroxocobalamin to Cyanocobalamin
Source: Nutrients. 2024 Jan 27;16(3):378. doi: 10.3390/nu16030378 (PMC10857013; doi:10.3390/nu16030378)
Supplement: Supplementary file 1 [file nutrients-16-00378-s001.zip › nutrients-2814181-supplementary.pdf]

Supplementary Materials

Kinetics of Cellular Cobalamin Uptake and Conversion: Comparison of Aquo/Hydroxocobalamin to Cyanocobalamin

Sergey N. Fedosov [1,2,*], Ebba Nexo [2] and Christian W. Heegaard [1]

[1] Department of Molecular Biology and Genetics, Aarhus University, 8000 Aarhus C, Denmark;

[2] Department of Clinical Medicine /Clinical Biochemistry, Aarhus University Hospital, 8200 Aarhus N, Denmark

* Correspondence: snf@mbg.au.dk or snfedosov1960@gmail.com

Section S1. Fusion of Several Intermediates into One "Pooled Metabolite" in Kinetic Models, Proof of the Concept

$$Y_1 \xrightarrow{k_{y1}} \boxed{Y_2 \xrightarrow{k_{y2}} Y_3 \xrightarrow{k_{y3}} Y_4} \begin{array}{l} \xrightarrow{k_{y45}} Y_5 \\ \xrightarrow[k_{y46}]{} Y_6 \end{array} \qquad \text{(S1)}$$

Fusion of several intermediates Y_i into a "pooled metabolite" ΣY_i can be illustrated using Equation (S1) as a convenient example. The scheme can be regarded as a full description of a reaction block, related to the uptake of TC-Cbl and including: Y_1 – extracellular TC-Cbl; Y_2 – absorbed TC-Cbl inside an endosome; Y_3 – TC-Cbl transferred to a lysosome; Y_4 – the liberated original vitamin-form transported to the cytoplasm; Y_5 – the original form of Cbl refluxed to the cell surface; Y_6 – the reduced form of Cbl remaining in the cytoplasm. All intermediates are connected by irreversible mass action reactions with the given rate constants k_{yi}. The intermediates Y_2, Y_3, and Y_4 (shown within a frame) cannot be distinguished from each other in our setup. Therefore, we present them as a "pooled" metabolite $\Sigma Y_i = Y_2+Y_3+Y_4$, which gives a new simplified layout (Equation (S2)) of the original "accurate" Equation (S1).

$$Y_1 \xrightarrow{k_{y1}} \boxed{\sum Y_i} \begin{array}{l} \xrightarrow{k_{25}^{app}} Y_5 \\ \xrightarrow[k_{26}^{app}]{} Y_6 \end{array} \qquad \text{(S2)}$$

Such layout cancels Y_2, Y_3, and Y_4 and the related rate constants k_{y2}, k_{y3}, k_{y45} and k_{y46}, but introduces instead a new intermediate ΣY_i and its new efflux coefficients: k_{25}^{app} and k_{26}^{app} (or just k_2^{app}, if only one efflux route exists). To connect the simplified Equation (S2) to the "true" Equation (S1), the canceled metabolite Y_4 (at the end of the pooled reactions) should be expressed via its fraction f_{Y4} in the sum of $Y_2+Y_3+Y_4$. Afterward, the new efflux coefficients k_{25}^{app} and k_{26}^{app} should be expressed via f_{Y4} stipulated via the set of canceled constants. In such way, Equation (S2) will closely imitate the efflux from Y_4 of Equation (S1). The approximate value of f_{Y4} can be assessed via a simple assumption. Thus, the metabolites of the central pool $\rightarrow(Y_2\rightarrow Y_3\rightarrow Y_4)\rightarrow$ remain for a considerable time in a pseudo steady-state ($dY_i/dt \approx 0$), being balanced by the influx (k_{y1}) and the efflux (k_{y45} and k_{y46}). Therefore, the below set of differential equations ((S3) – (S5), valid for Equation (S1)) can be used to express all constituents of the central pool (Y_2,Y_3,Y_4) via the same intermediate (e.g., Y_2) and to calculate f_{Y4}.

$$\frac{d[Y_3]}{dt} = k_{y2}\times[Y_2] - k_{y3}\times[Y_3] \approx 0; \qquad [Y_3] \approx \frac{k_{y2}}{k_{y3}}\times[Y_2] \qquad \text{(S3)}$$

$$\frac{d[Y_4]}{dt} = k_{y3}\times[Y_3] - k_{\Sigma4}\times[Y_4] \approx 0; \qquad [Y_4] \approx \frac{k_{y2}}{k_{\Sigma4}}\times[Y_2]; \qquad k_{\Sigma4} = \left(k_{y45}+k_{y46}\right) \tag{S4}$$

$$f_{Y4} = \frac{[Y_4]}{[Y_2]+[Y_3]+[Y_4]} \approx \frac{\left(k_{y2}/k_{\Sigma4}\right)\times[Y_2]}{\left(1+k_{y2}/k_{y3}+k_{y2}/k_{\Sigma4}\right)\times[Y_2]} = \frac{1/k_{\Sigma4}}{1/k_{y2}+1/k_{y3}+1/k_{\Sigma4}} \tag{S5}$$

The approximate value of f_{Y4} is quite accurate in the middle of the reaction progress, but is expected to deviate from the true fraction of Y_4 in the beginning and at the end of the process (i.e. when the intermediates of central pool are not in a steady state). Finally, the differential equation for Y_5 accumulation in Equation (S1) was transformed to include ΣY_i of Equation (S2) and to express k_{25}^{app} via k_{45} and f_{Y4}.

$$\frac{d[Y_5]}{dt} = k_{y45}\times[Y_4] \approx k_{y45}\times f_{Y4}\times[\Sigma Y_i] = k_{25}^{app}\times[\Sigma Y_i]; \qquad k_{25}^{app} = \frac{k_{y45}/k_{\Sigma4}}{1/k_{y2}+1/k_{y3}+1/k_{\Sigma4}} \tag{S6}$$

The analogous procedure for Y_6 gives the expression for k_{26}^{app}, where k_{46} substitutes for k_{45}, while the rest of equation remains identical to k_{25}^{app}. Proportion between Y_5 and Y_6 in Equation (S2) is determined by the ratio of k_{y45} / k_{y45}, as in Equation (S1). If branching to Y_6 is absent and the reactions follow a linear chain from Y_1 to Y_5, then the efflux equation (as well as its rate coefficient k_2^{app}) gets simplified, as shown in Equation S7.

$$\frac{d[Y_5]}{dt} = k_{y4}\times[Y_4] \approx k_{y4}\times f_{Y4}\times[\Sigma Y_i] = k_2^{app}\times[\Sigma Y_i]; \qquad k_2^{app} = \frac{1}{1/k_{y2}+1/k_{y3}+1/k_4} \tag{S7}$$

The aforementioned procedures connect the simplified Equation (S2) to the "true" Equation (S1), and one can expect a high degree of resemblance between the two models. Yet, some deviation is still expected, and the degree of this deviation would increase at a high representation of the pooled metabolites Y_2, Y_3 and Y_4 (i.e. the intermediates, whose concentrations cannot be mimicked with the absolute precision within Equation (S2)). Therefore, the behavior of a "faulty" Equation (S2) was modeled in respect to the "true" Equation (S1) on two examples. They represented (i) a difficult case, described by an "inconvenient" combination of constants (which give very high quantities of transient intermediates); and (ii) a more favorable case (where a reasonably "convenient" combination of constants gives moderate quantities of the transient intermediates). Reasonably adequate approximations were observed in both cases, see Figure S1a and Figure S1b. In the first case, a high quantity of Y_1+Y_2+Y_3 was accumulated, and the approximating tracks of ΣY_i and Y_5 (dashed lines) showed a somewhat higher deviation from the respective records of the "true" scheme (solid lines). In the second case (with a lower sum of Y_1+Y_2+Y_3) the deviation of the simplified scheme was less pronounced. We considered the presented material as a sufficient validation of our approach, where several metabolic steps and several intermediates are pooled together into a joined block.

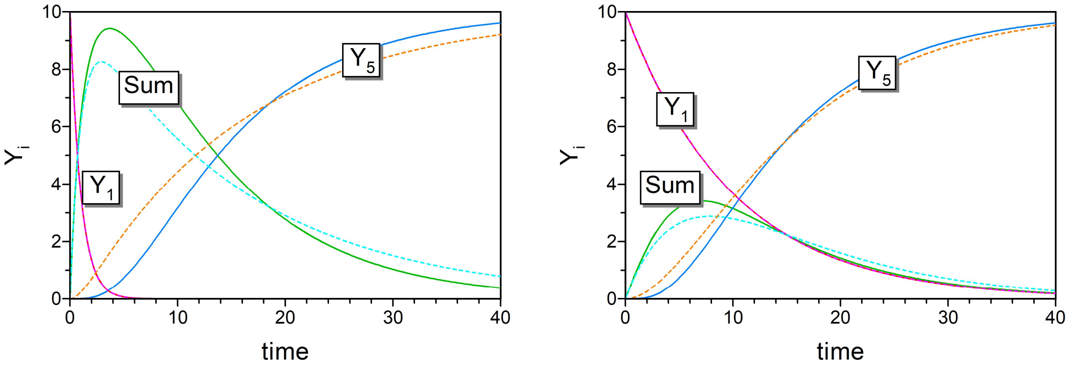

(**a**) (**b**)

Figure S1. Modeling of sequential conversions according to the "true" scheme $Y_1 \rightarrow Y_2 \rightarrow Y_3 \rightarrow Y_4 \rightarrow Y_5$ (solid lines) vs. its approximating imitation with pooled metabolites $Y_1 \rightarrow \Sigma Y_i \rightarrow Y_5$ (dashed lines).

The efflux constant of the simplified model (k_2^{app}) was calculated from the constants of the "true" scheme according to Equation (S7). All concentrations and rate constants are given in arbitrary units. The simulations were started from $Y_1 = 10$. The records for $Y_2+Y_3+Y_4$ or ΣY_i are notated as "Sum". (**a**) Modeling of a difficult case with an "inconvenient" combination of the true rate constants (k_{y1} = 1, k_{y2} = 0.3, k_{y3} = 0.5, k_{y4} = 0.1), corresponding to k_{y1} = 1 and k_2^{app} = 0.0652 in the simplified model. (**b**) Modeling of a favorable case with a "convenient" combination of the rate constants (k_{y1} = 0.1, k_{y2} = 0.3, k_{y3} = 0.5, k_{y4} = 1), corresponding to k_{y1} = 1 and k_2^{app} = 0.158 in the simplified model.

Section S2. Differential Equations used during Kinetic Modeling

The set of differential equation, which describe the kinetic scheme in Figure 5, is shown below.

$$\frac{d[R_{out}]}{dt} = v_1 - k_2 \times [R_{out}] - k_3 \times [R_{out}] \times [C_{out}] \quad \text{(S8)}$$

$$\frac{d[C_{out}]}{dt} = - k_3 \times [R_{out}] \times [C_{out}] + k_5 \times [C_{surf}] \quad \text{(S9)}$$

$$\frac{d[C_{in}]}{dt} = k_3 \times [R_{out}] \times [C_{out}] - (k_4 + k_6) \times [C_{in}] \quad \text{(S10)}$$

$$\frac{d[C_{surf}]}{dt} = k_4 \times [C_{in}] - k_5 \times [C_{surf}] \quad \text{(S11)}$$

$$\frac{d[C2]}{dt} = k_6 \times [C_{in}] - (k_{+7} + k_{+8}) \times [C2] + k_{-7} \times [MC] + k_{-8} \times [AC] \quad \text{(S12)}$$

$$\frac{d[MC]}{dt} = k_{+7} \times [C2] - k_{-7} \times [MC] \quad \text{(S13)}$$

$$\frac{d[AC]}{dt} = k_{+8} \times [C2] - k_{-8} \times [AC] \quad \text{(S14)}$$

Section S3. Validation of the Model by Analysis of Residuals

The accuracy of the model was examined by analysis of residuals. The verification process followed the steps listed below.

(C1) The average experimental error (standard deviation, σ) of the data was assessed in Figures 2a, 2b, 4a and 4b. For this purpose, the standard deviations (σ_i) were calculated for each set of points containing repeated measurements (time 24 h and 48 h in settings with CNCbl and HOCbl added). Tukey-Kramer tests (multiple pairwise comparisons) showed a high probability (p = 0.13 – 0.99) of an identical σ-value in these individual datasets in each particular panel. Therefore, the individual variances (σ_i^2) were averaged for each panel as follows:

$$\sigma^2 = \frac{\sum (n_i - 1) \times \sigma_i^2}{\sum (n_i - 1)}$$

where n_i is the number of points in the individual datasets (numbered as "i"). The calculated average σ-values are given in legends to Figures 2a, 2b, 4a and 4b.

(C2) Relative residuals (rr) were calculated for Figures 2a, 2b, 4a and 4b as $rr = (y_i - f_{(x)})/\sigma$, where y_i notates the experimental measurement, $f_{(x)}$ is the simulated theoretical value, and σ represents the respective experimental dispersion of the points in each particular panel, see the above paragraph (C1). Normality of the pooled rr-distributions in each panel was confirmed by Shapiro-Wilk test (p = 0.35 – 0.71).

(C3) Relative residuals (rr) in Figures 2a, 2b, 4a and 4b were examined as functions of time ($rr = P_1 \cdot t$) with the purpose of revealing a systematic deviation, expressed as the slope P_1 significantly different from zero. If such deviation was present, suitable parameters of the model were corrected. The final model gave the rr-chart shown in Figure S2 with the overall slope of $P_1 = -0.0010 \pm 0.0037$. Its identity to zero was highly probable (p = 0.79), as was confirmed by t-test. The model was assumed as a valid approximation of the experimental data.

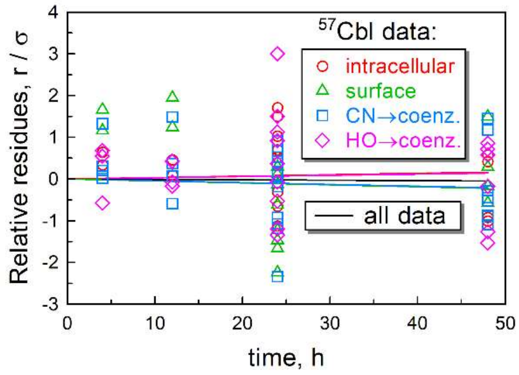

Figure S2. Dependences of relative residues (rr = r/σ) on time (rr = $P_1 \cdot t$). The calculated slopes were $P_1 = 0.00338 \pm 0.00744$ (Figure 2a, accumulation of the intracellular ^{57}Cbl, red circles); $P_1 = -0.00465 \pm 0.01058$ (Figure 2b, turnovers of the surface ^{57}Cbl, green triangles); $P_1 = -0.00435 \pm 0.00555$ (Figure 4a, conversion of CN^{57}Cbl to the Cbl-coenzymes, blue squares); and $P_1 = 0.00290 \pm 0.00780$ (Figure 4b, conversion of HO^{57}Cbl to the Cbl-coenzymes, magenta diamonds). The respective probabilities of a zero slope (i.e. absence of a systematic error in the model) were p = 0.656, 0.666, 0.440 and 0.714. The global fit of all relative residues (black line) gave $P_1 = -0.0010 \pm 0.00372$ (equal to zero with p = 0.79).

Section S4. Preservation of Cbls during Phenol-Chloroform Extraction

A mixture of several Cbls was added to either (i) test sample containing blood plasma + 10 mM glutathione (GSH), all diluted 1 : 5 with 0.32 M NH_4Acetate buffer, pH 4.6; or (ii) control sample containing 0.2 M NH_4Acetate buffer, pH 9.3. The Cbl-mixture included GSCbl, HOCbl, CNCbl, MeCbl, AdoCbl, the final concentration of each Cbl = 10 μM. The control sample was just heated before HPLC analysis (see Section 2.3), while the test mixture was extracted according to the full method described in Section 2.3. At the end of this procedure, the concentration of Cbls in the test sample (dissolved in 0.2 M NH_4Acetate buffer, pH 9.3) was adjusted to match Cbls in the control sample. Both preparations were subjected to HPLC analysis, and the profiles are compared in Figure S3. Both samples showed a successful conversion of GSCbl and HOCbl to NH_3Cbl. No change in the proportion of Cbls was found in the test sample compared to the control sample (compare green and blue profiles in Figure S3).

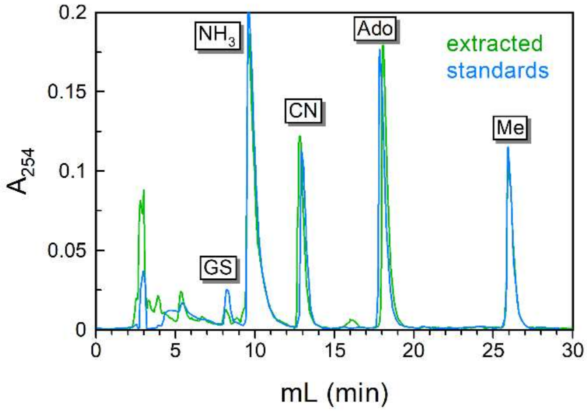

Figure S3. HPLC profile of the extracted Cbl-mixture (green record) compared to the control mixture (blue record). Notations GS, NH_3, CN, Ado, Me reflect the coordinated X-groups in each particular XCbl, eluted at the indicated retention time.
